# Assessing electrogenetic activation via a network model of biological signal propagation

**DOI:** 10.3389/fsysb.2024.1291293

**Published:** 2024-03-01

**Authors:** Kayla Chun, Eric VanArsdale, Elebeoba May, Gregory F. Payne, William E. Bentley

**Affiliations:** ^1^ Fischell Department of Bioengineering, University of Maryland, College Parko, MD, United States; ^2^ Institute for Bioscience and Biotechnology Research, University of Maryland, College Park, MD, United States; ^3^ Robert E. Fischell Institute for Biomedical Devices, University of Maryland, College Park, MD, United States; ^4^ Medical Microbiology and Immunology Department, University of Wisconsin-Madison, Madison, WI, United States

**Keywords:** network science, electrogenetics, synthetic biology, molecular information, microbial dynamics

## Abstract

**Introduction:** Molecular communication is the transfer of information encoded by molecular structure and activity. We examine molecular communication within bacterial consortia as cells with diverse biosynthetic capabilities can be assembled for enhanced function. Their coordination, both in terms of engineered genetic circuits within individual cells as well as their population-scale functions, is needed to ensure robust performance. We have suggested that “electrogenetics,” the use of electronics to activate specific genetic circuits, is a means by which electronic devices can mediate molecular communication, ultimately enabling programmable control.

**Methods:** Here, we have developed a graphical network model for dynamically assessing electronic and molecular signal propagation schemes wherein nodes represent individual cells, and their edges represent communication channels by which signaling molecules are transferred. We utilize graph properties such as edge dynamics and graph topology to interrogate the signaling dynamics of specific engineered bacterial consortia.

**Results:** We were able to recapitulate previous experimental systems with our model. In addition, we found that networks with more distinct subpopulations (high network modularity) propagated signals more slowly than randomized networks, while strategic arrangement of subpopulations with respect to the inducer source (an electrode) can increase signal output and outperform otherwise homogeneous networks.

**Discussion:** We developed this model to better understand our previous experimental results, but also to enable future designs wherein subpopulation composition, genetic circuits, and spatial configurations can be varied to tune performance. We suggest that this work may provide insight into the signaling which occurs in synthetically assembled systems as well as native microbial communities.

## 1 Introduction

Synthetic biology has enabled the production and sensing of biomolecules through the design, testing and implementation of genetic circuits. In addition to guiding complex biosynthesis processes for therapeutic and industrial applications (Mimee et al., 2015; Jiang and Zhang, 2016; Cao et al., 2020), these engineered systems hold potential to communicate with and guide synthetic consortia and even native biomes (Hwang et al., 2017). Recently, synthetic consortia have been developed for leveraging the diversity of multi population systems in ways that expand biosynthetic potential and increase metabolic efficiency (Dinh et al., 2020; VanArsdale et al., 2022; Zhao et al., 2022; Gwon et al., 2023). The interactions within these engineered communities rely on robust cascades of molecular communication that convey information between cells (Quan et al., 2016; Servinsky et al., 2016). As such, system designs need to consider not only the genetic circuits within “designer” cells, but the communication networks that tie them together (Terrell et al., 2021).

**FIGURE 1:**
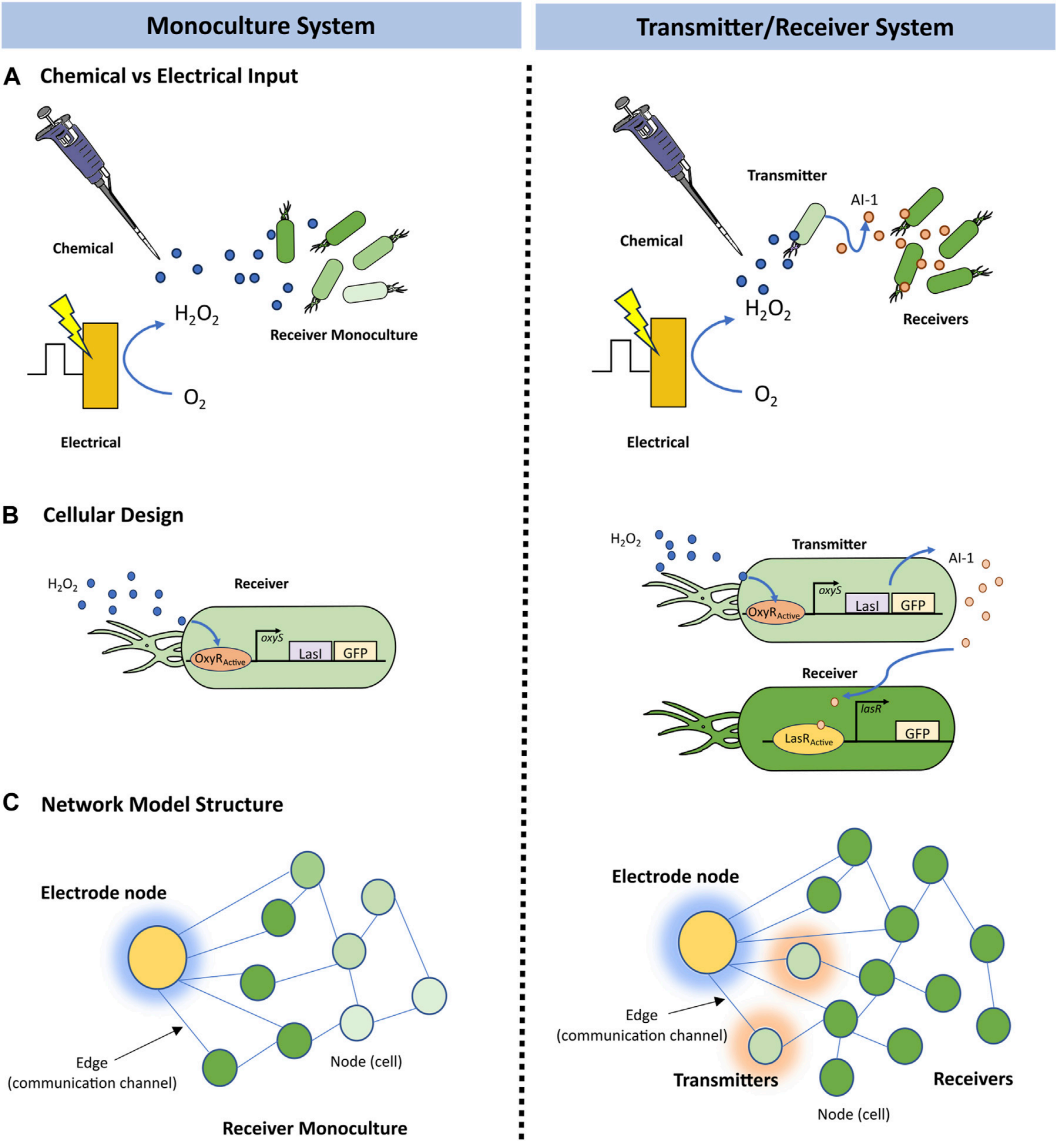
Systems overview. Schematic of the **(A)** cellular design of a Monoculture System (left) in which “Receiver” cells express LasI and GFP in response to hydrogen peroxide induction via the *oxyRS* regulon, and of a Transmitter/Receiver System (right) in which the same “Receiver” cells of the Monoculture system are repurposed as “Transmitter” cells that convey molecular information (AI-1 by the expression of *lasI*) to a second population also denoted “Receiver,” but that only express GFP in response to AI-1. **(B)** electrogenetic experimental setup where a biased gold electrode creates hydrogen peroxide as an initial input signal to the cellular systems, and **(C)** an example network model structure for the Monoculture and Transmitter/Receiver systems, saturation of green representing GFP levels and shading around nodes representing inducer production at those nodes.

In this study we wanted to mathematically characterize molecular signaling that guided previously published experimental results (VanArsdale et al., 2022) in which two cell-based systems synthesize a model product (green fluorescent protein, GFP) via chemical and electrical induction schemes exploiting different signaling pathways. These are depicted in Figure 1A. They are both based on induction by hydrogen peroxide. The base case (chemical induction) is actuated by the simple addition of hydrogen peroxide. Then, we had previously developed a means for electronically inducing cells; using simple electrodes, we altered the redox state of inducers (Tschirhart et al., 2017; Virgile et al., 2018; Kim et al., 2019; VanArsdale et al., 2022) and these activate genetic circuits. We refer to the genetic expression induced by electronic input as electrogenetics (Tschirhart et al., 2017) and have shown how one can electronically control gene expression, cell attributes (Virgile et al., 2018; VanArsdale et al., 2022), and even cell consortia (Tschirhart et al., 2017; Stephens et al., 2019; Bhokisham et al., 2020; VanArsdale et al., 2022; VanArsdale et al., 2023). In our experimental work (Figure 1A), we either added H_2_O_2_ (chemical induction) or we biased gold electrodes (2 mm diameter disk) immersed in the cultures with a—0.55 V vs. Ag/AgCl reductive potential (VanArsdale et al., 2022). This voltage is sufficient to electronically induce cells, it works by reducing oxygen dissolved in the growth media, creating hydrogen peroxide. Cells in the vicinity of the electrode genetically respond to the hydrogen peroxide through an engineered *oxyRS* regulon that activates a genetic circuit via the hydrogen peroxide sensitive transcriptional promoter, OxyR (Figure 1B). OxyR endogenously regulates oxidative stress management genes by repressing transcription until its cysteine groups are oxidized into disulfide bonds. The resulting conformation change stabilizes the transcription complex, inducing downstream gene expression (Pomposiello and Demple, 2001).

In Figure 1B, we illustrate the design of the two systems: (i) a receiver Monoculture and (ii) a Transmitter/Receiver co-culture. In the former case, hydrogen peroxide stimulates LasI and GFP production (Figure 1B). GFP is the model product in both cases and is easily measured by its fluorescence. LasI synthesizes the quorum sensing molecule N-3-oxo-dodecanoyl-L-homoserine lactone, which we refer to as autoinducer-1 (i.e., AI-1). Quorum sensing signaling molecules enable a collection of cells to take on a population-wide phenotype. In the Transmitter/Receiver system, the same cells used in the Monoculture system are repurposed as “transmitters,” where the hydrogen peroxide-induced quorum sensing signal is secreted and then encountered by the “receiver” cells and these respond by producing GFP (VanArsdale et al., 2022). Hence, in this two-strain culture one subpopulation turns the electronic signal into a biological signal for subsequent genetic activation and product synthesis in the second subpopulation. Autoinducer-1 is a very strong signaling molecule in that it activates gene expression at nanomolar amounts (Stephens et al., 2019). This amplifies the original signal to increase gene expression of the desired molecular product.

In this work, we employed a graphical modeling approach which enables a coarse grain interpretation of multicellular systems (Barabasi, 2013), thus, allowing us to capture agent-based intercellular interactions that fit population dynamics (Gosak et al., 2018). In Figure 1C, we depict our model in which each node represents a cell that possesses several weighted attributes: (i) local substrate concentration, (ii) the local inducer molecule concentration, and (iii) GFP expression level. The edges connecting nodes represent a communication channel where signaling molecules may transfer information between nodes. To characterize the movement of these signaling molecules, we implemented a previously developed overlay that approximates a formal diffusion model onto the network architecture (Sayama, 2015). This dramatically reduces computational demand while retaining dynamics of molecular communication and cellular connectivity.

With this model, we then characterized system performance in response to chemical and electrical induction by evaluating GFP production in both schemes. We further explored the effects of spatially fixed cultures (biofilms) in comparison to continuously stirred cultures by varying the edge dynamics in our model. Edges that are fixed reflect static cells, like would exist in a biofilm. Edges that are continually reconnecting between nodes reflect stirred cultures. Then, by utilizing modularity, a graph measure of a network’s subcommunity structure (Newman and Girvan, 2004), we related the network’s spatial organization to its signal output. Overall, our model enables a kinetic understanding of signal propagation and GFP production among spatially varied bacterial populations that, in turn, exploit different signaling processes. This provides new hypotheses regarding modes of information transmission and their effectiveness, ultimately leading to new designs.

## 2 Materials and methods

### 2.1 Model formalism

**FIGURE 2:**
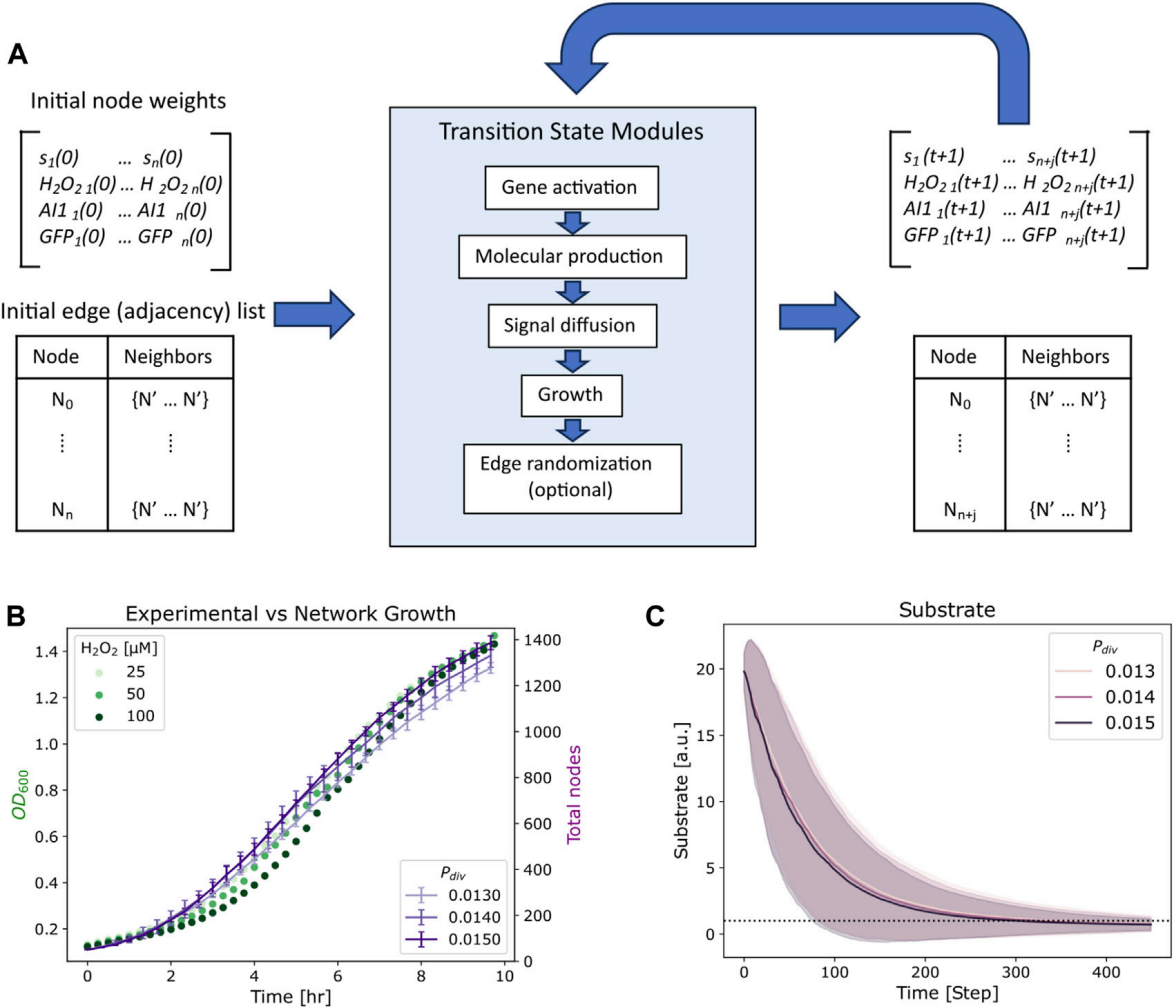
Simulation process and growth fit. **(A)** Overview of the simulation iterations, where initial state variables and edge structure are updated via transition state modules at each timestep. The output length of the state variables matrix and edge list increase by *j* new nodes. **(B)** Growth measurements for *E. coli* strain OxyR-LasI-GFP (transmitters) with various hydrogen peroxide induction concentrations (chemical addition) are plotted in green, alongside average total nodes of 10 simulation repeats at various division probabilities (*P*_*div*_) over time in purple. Bars represent standard deviation. **(C)** Average substrate per node for *P*_*div*_ in **(B)**, the horizontal dashed line indicates a user-specified substrate threshold, *k = 1*, below which a node will no longer divide. The shaded zones indicate standard deviation of the substrate concentration across the network for each probability.

Network initialization was performed by generating a random undirected G (n, m) graph (Barabasi, 2013) in which there are n total nodes and m total edges that are randomly distributed amongst the nodes. In this network, each node represents an individual cell and edges represent communication channels by which signaling molecules can be transferred between nodes. Each node N_i_ possesses the following dynamic node weights: *s*_*i*_(*t*), *H*_*2*_ *O*_*2*__*i*_(*t*), *AI-1*_*i*_(*t*)*,* and *GFP*_*i*_(*t*) corresponding to the cell’s substrate, hydrogen peroxide, autoinducer-1, and green fluorescent protein concentrations at time *t*, respectively. In this graph, edges are unweighted and undirected, meaning they do not possess quantitative attributes, nor do they follow any directionality in their connections, i.e., signaling molecules can flow to in either direction between two connected nodes. In our model, time is discrete and represented by natural numbers, evolving forward with each iteration of the simulation as depicted in Figure 2A. At each timestep a transition is applied in which each attribute of the network is sequentially updated via the following modules: (i) Gene activation, (ii) Molecular production, (iii) Signal diffusion, (iv) Growth, and (v) Edge randomization. That is, a gene activation module is applied, and then activated nodes carry out their respective molecular production models, resulting in increased molecular concentrations at these nodes. Next a signal diffusion module is applied, and molecular concentrations are updated based on the calculated exchange of molecules. Lastly, a growth module is applied to nodes with available substrate; the concentrations of divided nodes are amended. After this, edge randomization may be applied to stirred culture simulations, and the time is forwarded to the next timestep. Thus, the state of the system can be described at any point by the nodes, each with their own set of state variables described by their weights and edges as depicted in Figure 2A.

#### 2.1.1 Gene activation and molecular production

To capture genetic induction and subsequent molecular production we implemented a two step mechanism at each node at every timestep. First, the probability of gene activation is a function of inducer concentration (see Table 1; Equations 1–6). H_2_O_2_ induced gene expression is described by a logistic curve (Table 1; Equations 1, 2; Supplementary Figure S1A) with a threshold of 12.5 µM. AI-1 dependent gene expression is implemented using a steeper and linear step function (Table 1: Equations 3, 4; Supplementary Figure S1A) reflecting the nanomolar requirements for induction (Chun et al., 2021; VanArsdale et al., 2022).

**TABLE 1:** Equations for gene activation and subsequent protein production for the inducers: hydrogen peroxide and AI-1.

Description	Equation
Hydrogen peroxide induced gene activation probability	(1) ${Prob}_{H_{2}O_{2}}\left(H_{2}O_{2}>0\right)=\frac{1}{1+e^{-\frac{{\left[H\right.}_{2}O_{2}]-8}{2}}}$
	(2) ${Prob}_{H_{2}O_{2}}\left(0\right)=0$
AI-1 induced gene activation probability	(3) ${Prob}_{AI1}\left(AI1>0\right)=\frac{1}{1+e^{-50\left(\left[AI1\right]-0.25\right)}}$
	(4) ${Prob}_{AI1}\left(0\right)=0$
Hydrogen peroxide induced molecular production rate	(5) ${Rate}_{H2O2}=\frac{2\left[S\right]}{1+e^{\left.-{\left[H\right.}_{2}O_{2}\right]}}$
AI-1 induced molecular production rate	(6) ${Rate}_{AI1}=0.2\left[AI1\right]\left[S\right]$

If a gene is activated at a node for a timestep (via probability function based on inducer concentration), it will produce the specified molecular product (GFP or AI-1) at a set expression rate based on the prevailing inducer concentration and substrate availability (Table 1: Equations 5, 6; Supplementary Figures S1B, C). For production based on AI-1, the rate is linear while for hydrogen peroxide it is a saturation function so that at low concentrations there is a steep peroxide dependence and at high concentrations the rate is saturated (Stephens et al., 2019; Terrell et al., 2021; VanArsdale et al., 2022). These transitions occur at each timestep prior to the diffusion and growth modules, such that molecular production occurs with the concentrations from the previous timestep. The discrete equations are described in Table 2.

**TABLE 2:** State variable dynamics equations.

Variable	Equation
*H*_*2*_ *O*_*2*_	*H*_*2*_ *O*_*2*__*i*_(*t+1*) *=* $H_{2}O_{2\;i}\left(t\right)+\alpha \left[\sum_{j\in N_{i}}{H_{2}O_{2}}_{j}\left(t\right)-H_{2}O_{2\;i}\left(t\right)\deg\;\left(i\right)\right]$
*AI-1*	*AI-1*_*i*_(*t+1*) *=* ${AI1}_{\;i}\left(t\right)+{Prob}_{H_{2}O_{2}}*{Rate}_{H_{2}O_{2}}+\alpha \left[\sum_{j\in N_{i}}{AI1}_{j}\left(t\right)-{AI1}_{i}\left(t\right)\deg\;\left(i\right)\right]$
*s*	*s*_*i*_(*t+1*) *=* $\frac{s_{\;i}\left(t\right)}{2}$ (if node *i* divides)
*GFP*	*GFP*_*i*_(*t+1*) *=* ${Prob}_{AI1}*{Rate}_{AI1}+{GFP}_{\;i}\left(t\right)$ (AI-1 induced) or *GFP*_*i*_(*t+1*) *=* ${Prob}_{H_{2}O_{2}}*{Rate}_{H_{2}O_{2}}+{GFP}_{\;i}\left(t\right)$ (H_2_O_2_ induced)

#### 2.1.2 Signal diffusion

Signaling between nodes occurs across edges, such that only nodes connected by an edge may transfer H_2_O_2_ and AI-1. Signal molecule movement across edges are defined by a discrete approximation of diffusion derived by the following equations as previously described by Sayama (Sayama, 2015):

$$\frac{dc_{i}}{dt}=\alpha \sum_{j\in N_{i}}\left(c_{j}-c_{i}\right)$$ (1)

$$c_{i}\left(t+∆t\right)-c_{i}\left(t\right)=\left[\alpha \sum_{j\in N_{i}}\left(c_{j}-c_{i}\right)\right]∆t$$ (2)

$$c_{i}\left(t+∆t\right)=c_{i}\left(t\right)+\alpha \left[\sum_{j\in N_{i}}c_{j}\left(t\right)-c_{i}\left(t\right)\deg\;\left(i\right)\right]∆t$$ (3)

where *c*_*i*_ is the concentration of signaling molecule at a given node *i*, *c*_*j*_ is the concentration at that node’s neighbor *j*, *deg*(*i*) is the number of edges at node *i,* and *α* is a diffusion coefficient (See Supplementary Table S1 for all coefficient values). In Eq. 1, diffusion is generalized to the change in concentration at a node with respect to the difference between its own concentration and its neighbors. This can be discretized (Eq. 2) and solved to find that the change in concentration at a node is determined by the difference between the sum of its neighbors’ concentrations and the product of its own concentration and number of edges (Eq. 3). At every timestep, the concentration is calculated from Eq. 3 for each node and updated prior to growth module implementation. This process applies to the following state variables and occurs prior to the calculation of network growth: *H*_*2*_ *O*_*2*_ (*t*) and *AI-1*(*t*). The equations for these variables prior to network growth can be found in Table 2.

#### 2.1.3 Network growth

The network grows with time, depending on substrate availability and growth probability, *P*_*div*_. Initially, each node is assigned the same initial substrate weight, *s*_*0*_. At each time step, if a node has a substrate level above a minimum threshold, *k*, the node has the probability *P*_*div*_, that it may divide into two. Following a division event, the substrate (Table 2), H_2_O_2_ and AI-1 node weights are divided equally between daughter nodes at each timestep. As noted above this occurs after the diffusion module, such that the newly calculated H_2_O_2_ and AI-1 concentrations may be divided in two upon a division event. As depicted in Figure 2A, with each iteration the network will increase by *j* nodes, determined by substrate availability at each node and *P*_*div*_. We note that daughter nodes maintain fluorescence (GFP) of their parent’s. This assumption is in agreement with previous experiments (Servinsky et al., 2016). We additionally neglect protein degradation, again in agreement with experimental results (Servinsky et al., 2016).

After a division event, the resulting daughter nodes share an edge and maintain their parent’s edges, limited to a maximum of 10 neighbors. Note, as commonly defined within the field of network science, we refer to neighbors as nodes which share an edge (Newman et al., 2006). These 10 neighbors are randomly sampled from the parent’s neighbors including those that have divided at that timestep. In a case where a dividing node has 10 neighbors that also all divide at that time step, out of the 20 surrounding nodes only 10 will be randomly selected to share an edge with each daughter.

In Figure 2B, we depict growth curves for the *Escherichia coli* strain OxyR-LasI-GFP grown with various hydrogen peroxide concentrations (VanArsdale et al., 2022). These cells are the receivers in the Monoculture case and transmitters in the Transmitter/Receiver case (Terrell et al., 2021) (Figure 1). Alongside we show the total number of nodes over time for a simulated network of with 50 initial nodes, an *s*_*0*_ of 20, and a *k* of 1 for various *P*_*div*_. With these *s*_*0*_ and *k* values, each node can divide five times during the growth phase, allowing us to fit the initial node count to 50 and total possible number of nodes to 1,600 which approximates 1 node to 0.001 OD_600_. The *P*_*div*_ values assigned helped to ensure that the growth phase of the network translated well to experimental results, such that 45 timesteps represented ∼1 h of cell culture. Our simulation thus mimicked the log phase growth of the cell cultures. We note that flexibility for fitting experiments is enabled by altering the division probability, *P*_*div*_. Additionally, we note that as the network grows the average substrate per node decreases over time (Figure 2C), until reaching below the threshold value of *k =* 1. As described above, below this threshold, nodes may no longer divide. The shadowed area in Figure 2C represents the full ranges of substrate levels across the network and for each division probability. While the substrate defined in our model represents general nutrient availability, the trend shown in Figure 2C emulates the decrease in glucose over time in *E. coli* cultures demonstrated experimentally (Shiloach et al., 1996). That is, while the network model formalism does not include a typical deterministic Monod model for growth with a maximum specific growth rate and saturation constant, the configuration here well represents the overall culture dynamics.

#### 2.1.4 Edge randomization

To describe the spatiotemporal effects of various modes of cell culture such as stirred, immobilized biofilms (static), and combinations thereof, we implemented edge randomization. In the absence of stirring, edges which are assigned during network initialization and at each node division, remain fixed for the duration of a simulation. To simulate a stirred batch culture, we randomized the edges amongst all nodes at every timestep. We simulated two base cases with either static or randomized edges and with or without network growth to demonstrate the effects signaling dynamics: one case where inducers may come from a highly concentrated source node and another case where an electrode may generate inducers at its surface over a specific time period (Supplementary Figure S2). As anticipated, cases that include network growth and edge randomization resulted in faster homogeneity of signaling molecule concentration across the network than non-growing networks or those growing with static edges. From these tests, we found a set of parameters that when used, enabled reasonable agreement between our previously published data (*s*_*0*_ = 20, *k =* 1, *P*_*div*_ = 0.015, *α* = 1 and an initial average of 4 edges per node).

#### 2.1.5 Electrical hydrogen peroxide generation

To mathematically characterize the production of hydrogen peroxide at the surface of a biased electrode as a mode of information transmission into bacterial cells, we model the input as a signal generated from an individual source node, then link this source to the various nodes. In our network architecture, the electrode is represented by a single node which produces hydrogen peroxide at each time step that it is turned “on.” To simulate the actual experimental conditions in which electrical stimulus resulted in negligible growth during the time of induction (VanArsdale et al., 2022), we set the growth probability parameter, *P*_*div*_, to zero when the electrode is “on” until that time when the growth was observed to increase. We fit the hydrogen peroxide production for an initial network size of 100 to produce 46 µM hydrogen peroxide per timestep to approximate experimental results (Supplementary Figure S3A).

Previously reported experimental results demonstrated that electrical induction yielded lower GFP output compared to a chemical addition, suggesting that the spatiotemporal heterogeneity resulting from the localized inducer production at the electrode’s surface effects output. To recapitulate these findings in our model we limited the number of nodes connected to the electrode to 5% of the total network at every timepoint. In Supplementary Figure S3B, we plotted the Monoculture response for chemically and electrically induced simulations to demonstrate that the limitation of electrode connectivity to the network reproduces experimental trends, via reduced GFP production compared to chemical induction.

### 2.2 Code and data availability

Graph simulations were performed in Python using NetworkX (Hagberg et al., 2008), and modifying and implementing the Simulation class from A First Course in Network Science (Menczer et al., 2020). Graph generation and initialization and graph transition states were defined and are contained in supplemental notebooks. Visualizations were performed using Python’s matplotlib and seaborn libraries (Hunter, 2007; Waskom, 2021). Experimental data used for parameter fitting are from (VanArsdale et al., 2022).

Python notebooks and simulation data are available online at github.com/kaychun29/bio-network-simulations.

## 3 Results

### 3.1 Chemical and electrical induction of monoculture and transmitter/receiver systems

**FIGURE 3:**
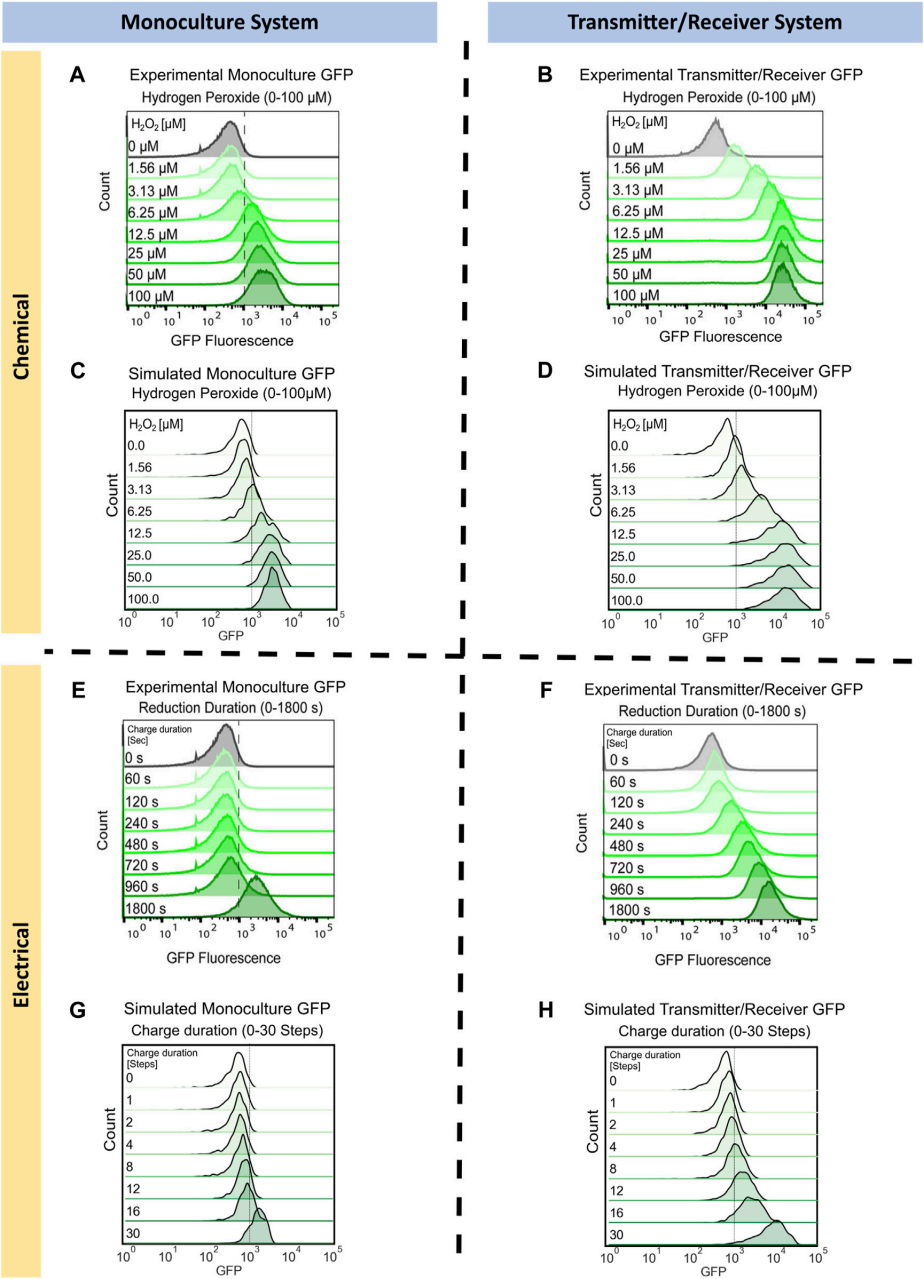
Monoculture and Transmitter/Receiver GFP distributions for chemically and electrically induced edge randomized networks. Chemically induced **(A)** Monoculture and **(B)** Transmitter/Receiver system at 3 h hydrogen peroxide addition, reproduced with permission from VanArsdale et al. (2022). **(C)** The simulated monoculture system GFP distribution at 180 timesteps is shown for an aggregate of 10 replicates, with initial hydrogen peroxide concentration ranging from 0 to 100 µM. **(D)** The simulated Transmitter/Receiver system’s GFP distribution across all nodes at 180 timesteps is shown for an aggregate of 10 replicates, with initial hydrogen peroxide concentration ranging from 0 to 100 µM. Experimental flow cytometry data of the **(E)** Monoculture and **(F)** Transmitter/Receiver system at 3 h post charge application, reproduced with permission from VanArsdale et al. (2022). GFP distributions of simulated electrical induction for the **(G)** Monoculture system and **(H)** Transmitter/Receiver’s receiver GFP distributions across all nodes at 180 timesteps post charge application. Distributions shown are an aggregate of 10 simulated replicates, with charge durations ranging from 0-30 timesteps.

We first simulate the two cellular systems in response to the chemical addition of hydrogen peroxide. We aimed to capture the experimental results depicted in Figures 3A, B (reproduced with permission), where identical levels of hydrogen peroxide were added to the Monoculture system and to the Transmitter/Receiver System. We later measured GFP expression in all cells via flow cytometry after 3 h (VanArsdale et al., 2022). Flow cytometry provides for the distribution of GFP among all cells in a population. Especially at high concentrations, a chemical addition of hydrogen peroxide should result in a homogeneous input (VanArsdale et al., 2022) wherein there is little “noise” accompanying induction. In the Monoculture system, increases in GFP became obvious at initial concentrations of 12.5 uM H_2_O_2_. Further increases in H_2_O_2_ had relatively little effect on GFP. Interestingly, for the Transmitter/Receiver system, lower initial concentrations of H_2_O_2_ resulted in significant GFP expression owing to the AI-1 signal propagation. In the end, the yield of GFP for this Transmitter/Receiver system was nearly 10-fold higher than the case with just H_2_O_2_ added to the monoculture, even at the highest concentrations (VanArsdale et al., 2022).

To simulate these results, we assigned each node the same initial hydrogen peroxide weight based on the initial experimental concentration. We set initial GFP weights randomly using a Gaussian distribution with a mean of 500 and standard deviation of 250. This allows for all nodes to have fluorescence background, which fit our previously published experimental distribution for uninduced cells, Figures 3A, B (VanArsdale et al., 2022). For the following simulations the initial network size was 100 nodes, with an average of four edges per node. These initial conditions enabled reproducible network propagation, while conserving computational time. We implemented network growth and edge randomization at each timestep to recapitulate the well-mixed growing culture, according to the methods previously described. For the Monoculture system, chemical induction was simulated using the gene activation probability ( ${Prob}_{H_{2}O_{2}}$ , Table 1: Equations 1, 2) and the molecular production rate ( ${Rate}_{H2O2}$ , Table 1: Equation 5). To model the Transmitter/Receiver system in which a two-strain co-culture is used to amplify the initial hydrogen peroxide signal, we partitioned the initial network into 10 percent transmitter nodes, which function the same as the Monoculture’s receivers, and 90 percent receiver nodes which activate GFP production by AI-1 induction. In both systems, AI-1 freely diffuses between nodes at each timestep (Stephens et al., 2019), while in neither case does the GFP diffuse out of the cell^47^. In this Transmitter/Receiver system, GFP production is probabilistically activated ( ${Prob}_{AI1}$ , Table 1; Equations 3, 4) and produced at a rate ( ${Rate}_{AI1}$ , Table 1: Equation 6) dependent on AI-1 and substrate concentration. In Figures 3C, D we plotted the simulated GFP distributions across the entire network for both the Monoculture and Transmitter/Receiver networks at mid-log growth (180 timesteps) as a function of initial H_2_O_2_ level. Consistent with the experimental results, the range of expression in the Transmitter/Receiver network reached 10^5^, while the Monoculture network’s maximum values were ten-fold lower.

We next simulated the electrogenetic approach wherein an applied reducing potential on the electrode generates hydrogen peroxide and this, in turn, stimulates the cells. Naturally, a major difference between this mode of induction is that the hydrogen peroxide is generated at the electrode and while the system is mixed, the peroxide level increases with the extent of its generation rate. The experimental results from earlier work are shown in Figures 3E, F (reproduced with permission) (VanArsdale et al., 2022). In the Monoculture system, small increases in GFP were observed until the cells were exposed to −0.55 V for 1,800 s. In the previous work, a solution exposed to this reduction duration produced approximately 15 µM of H_2_O_2_ (VanArsdale et al., 2022). Thus, the experimental results for the electrogenetic case were roughly equivalent to the chemical addition of H_2_O_2_. It was interesting to see that in the case of the Transmitter/Receiver system, a continuous increase in GFP was observed with increased charge. This was previously described as a result of cells near the electrode experiencing sufficient peroxide to induce AI-1, which, in turn, is stable and can be mixed throughout (VanArsdale et al., 2022).

To simulate electrical induction, we utilized the same model structure as described prior for chemical induction with the exception of initial hydrogen peroxide concentrations. For electrical induction, initial hydrogen peroxide weights were set to zero across the whole network and hydrogen peroxide was produced over a designated charge duration as described in **Methods**. In Figure 3G, we found the simulated GFP distribution of the electrically induced Monoculture system did not increase significantly until greater than 30 timesteps of applied charge (equivalent of 30 min), aligning with experimental results in Figure 3E. For the Transmitter/Receiver system (Figure 3H), activation increased nearly immediately, and full activation was attained with 30 steps of electrode charge. Our network model, in all cases, corresponded well with the actual data in Figures 3E, F, wherein the Monoculture distribution was essentially unchanged until over 960 s and the Transmitter/Receiver distribution increased across the span of 960 s to reach full activation.

**FIGURE 4:**
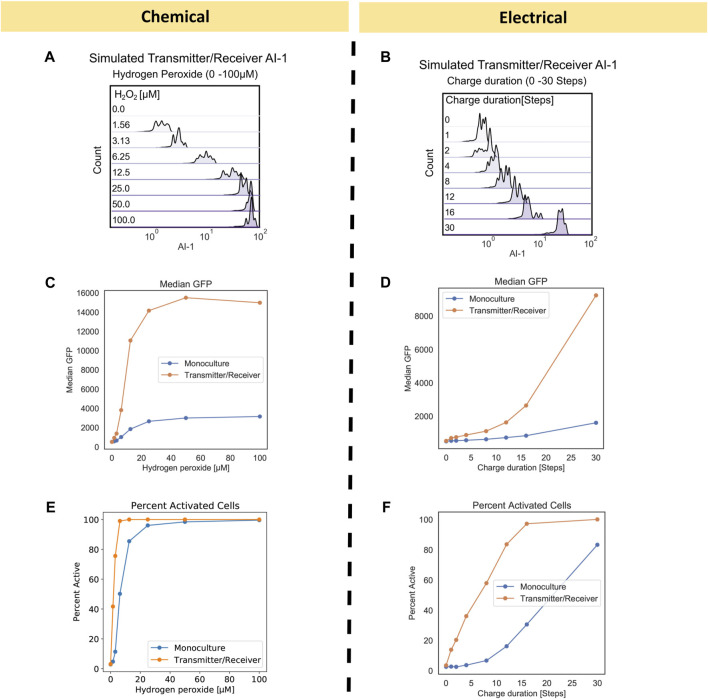
Transmitter/Receiver AI-1 distributions and signal metrics of chemically and electrically induced edge randomized networks. **(A)** The AI-1 distribution amongst all nodes in the Transmitter/Receiver network at 180 timesteps is shown for an aggregate of 10 replicates. **(B)** The AI-1 distribution amongst all of nodes in the Transmitter/Receiver network at 180 timesteps post charge application is shown for an aggregate of 10 replicates. **(C, D)** Calculated median GFP from the distributions data shown in Figure 3 plotted over their initial inducer concentration **(C)** and charge duration **(D)**. **(E, F)** Calculated percent active nodes from the distributions data shown in Figure 3 plotted over their initial inducer concentration **(E)** and charge duration **(F)**, threshold for activation was defined at 1000 GFP.

An advantage of the network approach is that one can examine state variables that are otherwise difficult to obtain experimentally. Also, one can more easily align results with underlying mechanisms. In Figures 4A, B, we plotted the estimated AI-1 distributions for the Transmitter/Receiver networks. While not measured experimentally (VanArsdale et al., 2022), these simulated values are consistent with expectations. The AI-1 distributions suggest significant heterogeneity within the network. We found this heterogeneity was a result of the variance in activation and spatial distribution of the transmitter nodes and we note this heterogeneity has been reported in chemically induced bacterial cell cultures (Servinsky et al., 2016). We also note that such heterogeneity is not characterized with commonly implemented population scale ODE models, but it can be manipulated experimentally via quorum sensing and genetic circuit design (Zargar et al., 2015). Our initial network model suggests that there is a level of heterogeneity that is innate to the system and is introduced when amplifying an initial homogenous input through a subpopulation of cells.

Interestingly, we found that the range of GFP for both Transmitter/Receiver systems was reflected in the AI-1 distributions in Figures 4A, B **.** In the chemically induced system, the AI-1 concentrations were between 10^1^–10 (Jiang and Zhang, 2016) for initial H_2_O_2_ concentrations above 6.25 µM. Comparatively, for the electrically induced system the AI-1 distribution across the entire network increased incrementally with only the highest charge duration of 30 timesteps producing above 10^1^ of AI-1. We further evaluated signal transmission by assessing the median GFP and fraction of activated cells for chemical and electrical induced systems. These serve as metrics for final signal output. The median GFP shows that with electronic induction, expression was generally lower than with chemical induction (Figures 4C vs. 4D), suggesting the signal was attenuated when the inducer was produced at a point source (the electrode node) and needed to diffuse outward among the cells to provide induction.

When comparing the Monoculture to Transmitter/Receiver systems, we observed the amplified response enabled by the Transmitter/Receiver system was readily apparent; the median GFP was above 1.4 × 10^4^ *versus* 2.5 × 10^3^ for the Monoculture (Figure 4C), an approximate 5-fold increase, when chemically induced with 100 µM. With electrical induction the median GFP of the Transmitter/Receiver system reached about 8.5 × 10^3^ at the longest charge duration (30 steps), whereas the Monoculture system did not increase above 2.0 × 10^3^, an approximate 4-fold difference. In addition to median GFP we also calculated the percent activated nodes in the network for each initial inducer concentration (by measuring the number of nodes with GFP above a 10^3^ threshold). In Figure 4E, we plotted chemically induced systems and observed that although both systems ultimately reached 100% activity, the Transmitter/Receiver system reached this peak at lower H_2_O_2_. For the electrically induced systems, the portion of active nodes increased incrementally and monotonically with charge (Figure 4F). We note that the Monoculture system had a consistently lower percentage of active nodes than the Transmitter/Receiver system, as expected, and never reached 100% by with 30 timesteps of induction. Overall, our model simulations corresponded well with the previous data (Figures 3A, B, E, F). Our simulations also suggest that despite the heterogeneity or “noise” that is introduced by amplifying the initial signal through a subset of cells (electrode induction), the molecular amplification that was enabled by transforming the H_2_O_2_ into a stronger secondary signaling molecule, in particular one that evokes a quorum sensing response, overcame that disruption, and produced high levels of signal and activation.

**FIGURE 5:**
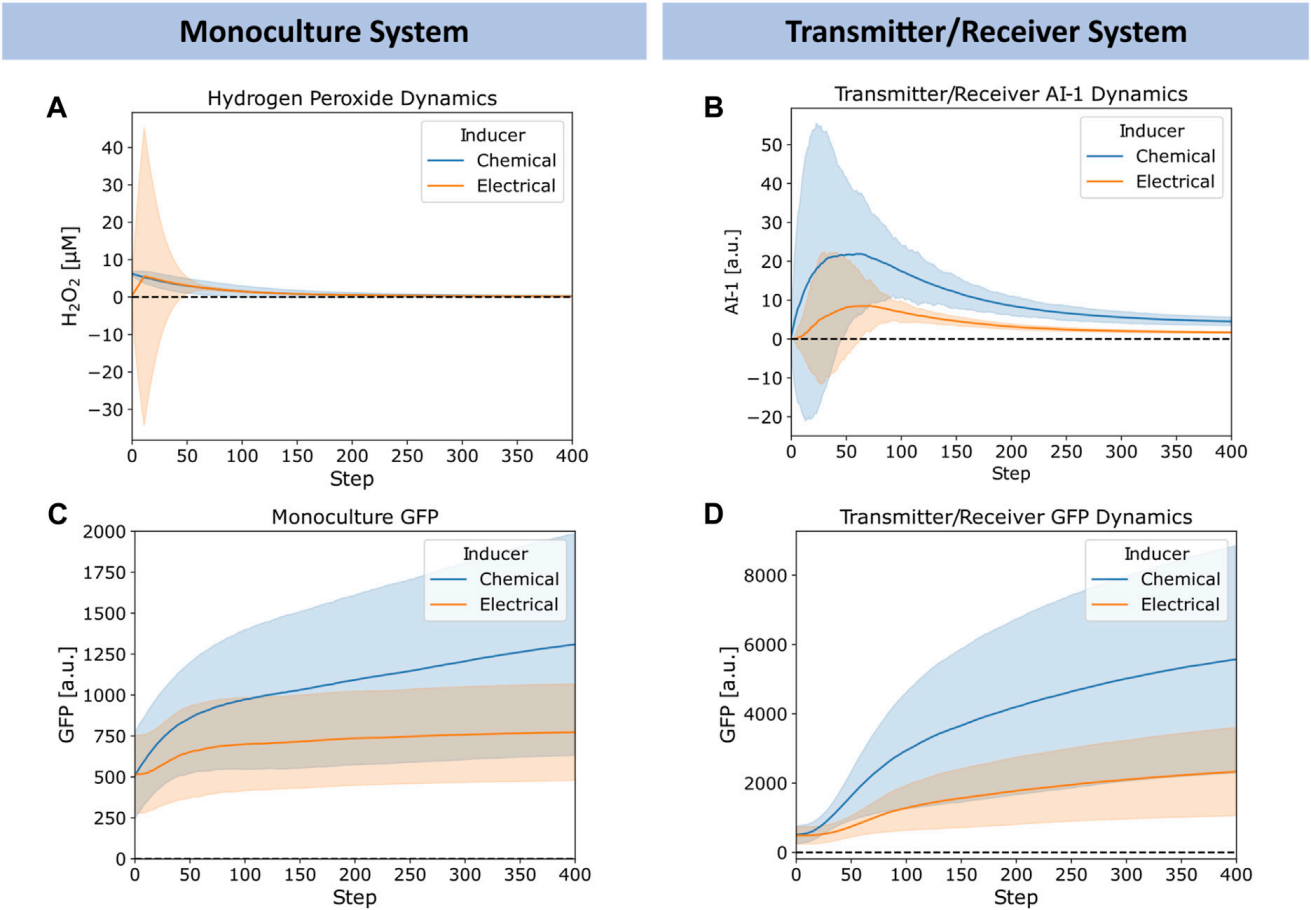
Chemical *versus* electrical signaling dynamics for edge randomized networks. **(A)** Average hydrogen peroxide, **(B)** Average Monoculture GFP, **(C)** Average Transmitter/Receiver GFP, and **(D)** Average Transmitter/Receiver AI-1 concentrations over time for a 6.25 µM hydrogen peroxide induced chemical addition (blue) and 12 step charge duration (orange) across the entire network. Error bars appear as shaded regions, representing standard deviation of aggregated network data from 10 simulation replicates.

In Figure 5, we explored further the dynamics of H_2_O_2,_ AI-1, and GFP for the chemically and electrically actuated cases by plotting their average (lines) and standard deviation (shaded) across the network over time. We chose representative cases with similar average H_2_O_2_ concentrations. In Figure 5A, we depict the simulated H_2_O_2_ dynamics for the chemical addition of 6.25 μM H_2_O_2_ and for the electrical induction at 12 timesteps of applied charge (∼6 µM of hydrogen peroxide generated). The widely distributed H_2_O_2_ level in the case of electrical induction was expected, but the average concentration simulated was quite similar. We note, Figure 5A depicts Transmitter/Receiver H_2_O_2_ dynamics, however Monoculture dynamics were nearly identical suggesting the type of cellular system does not affect hydrogen peroxide diffusion and generation. Despite the comparable average H_2_O_2_ levels in the systems over time, the AI-1 concentration of the Transmitter/Receiver system was nearly 2-fold higher that of the chemical induction (Figure 5B). In general, the GFP levels produced by both the Monoculture and Transmitter/Receiver systems (Figures 5C, D) were higher for the chemical addition relative to the electronically induced systems. This was understandable because the electrode produced H_2_O_2_ levels were found to be widely dispersed, indicating that many cells likely encountered minimal levels of inducer (Figure 5A). When comparing the Monoculture *versus* Transmitter/Receiver GFP dynamics (Figure 5C vs. Figure 5D), GFP expression in the Monoculture increased consistently over time whereas the Transmitter/Receiver network expression was slightly delayed initially during which time AI-1 was produced (∼50 steps corresponding to peak AI-1) and subsequently accumulated. For both modes of induction, the Transmitter/Receiver GFP yields were higher irrespective of a delay in production.

Overall, we note that the large standard deviations depicted in Figure 5 reflect substantial heterogeneity within the network. We suggest this heterogeneity is rooted in the wide signaling molecule distribution that can occur when cell numbers are low (early on) and when electrodes are used to generate hydrogen peroxide. In the latter case, this signal molecule interacts with cells in a random and distributed manner. In the experimental system, an uninduced cell needs to be transported near an electrode to receive H_2_O_2_. At further distances the peroxide could be depleted so that cells far away never experience high levels. Interestingly, our network model seems to well characterize the extent of signal propagation and the effects of its design structure in determining system outcome. The tradeoffs between the delay in responses and expression levels provide insight on system design. They also suggest spatial heterogeneity, we explore this as a potential design feature as follows.

### 3.2 Spatial design via network topology: Graph modularity and edge dynamics’ effect on signaling

**FIGURE 6:**
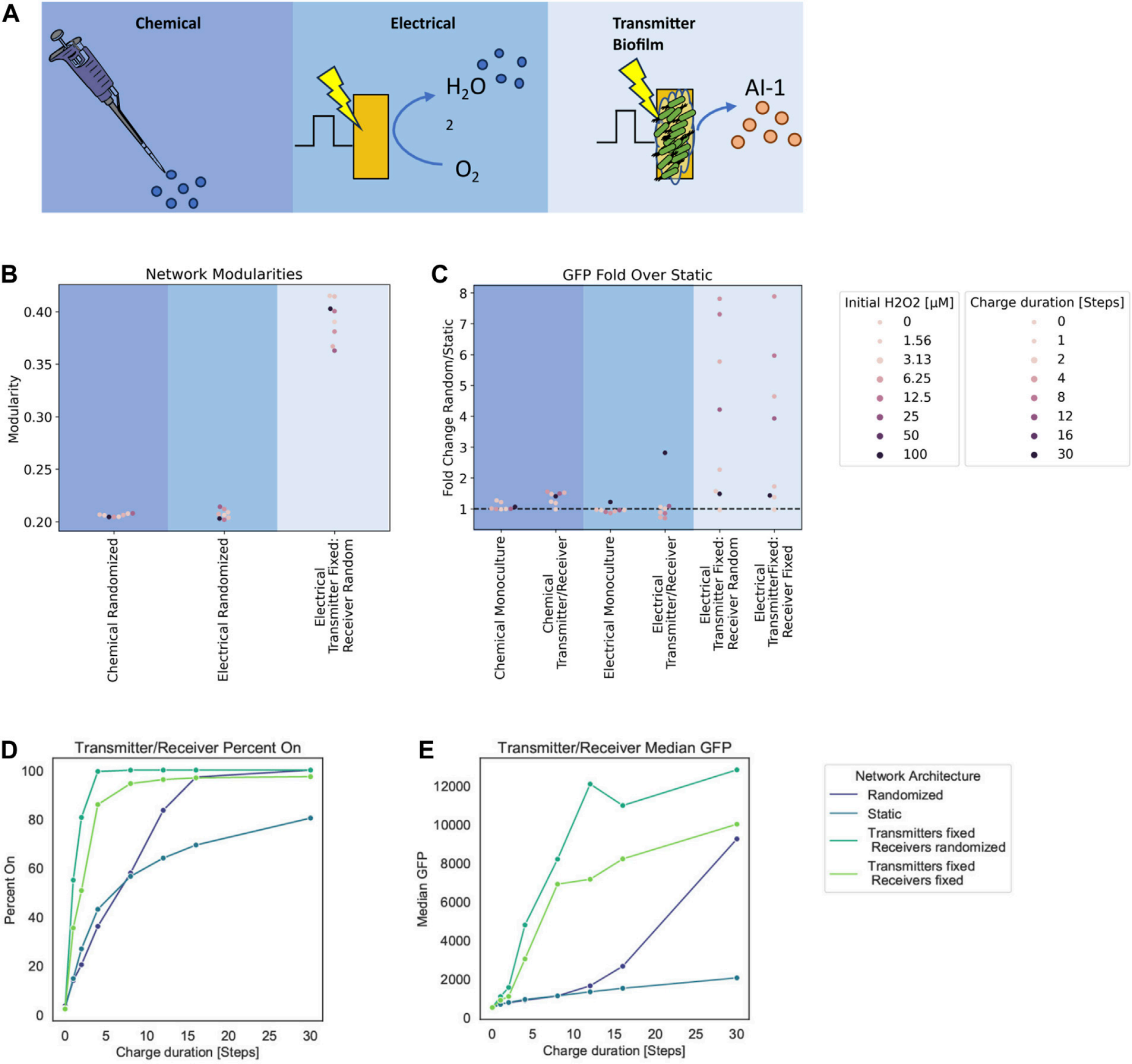
Modularity and fold change dependent on network structure. **(A)** Graph schematic of the three spatial configurations tested. **(B)** Network modularity of differing node arrangements and edge dynamics at timestep 180. **(C)** Fold change in GFP of randomized edge networks over static edge networks for the Monoculture and Transmitter/Receiver systems with either chemical or electrical induction at 180 timesteps post induction. **(D, E)** Signal transmission metrics for Transmitter/Receiver network architectures at 180 steps post 30 steps of electrical induction, calculated from an aggregate distribution of 10 simulation replicates. **(D)** Percent active nodes for varying charge duration times. **(E)** Median GFP across network for varying charge duration times.

Based on our successes in characterizing experimental data from both the chemical addition of hydrogen peroxide and its electrode-based generation for both the Monoculture and the Transmitter/Receiver systems, we decided to interrogate the design space for altered induction methodologies. Specifically, we next explored how the relative spatial distribution of cells (nodes) could affect the signaling. We decided to test a case where we retain transmitter cells directly onto the electrode. Thus, in Figure 6A, in addition to the (i) a chemical addition and (ii) electrical induction cases previously described, we added (iii) electrical induction of transmitter cells that are fixed to its surface. This last network structure captures experimental designs in which cells are either engineered to bind to gold electrodes (Terrell et al., 2021) or that are retained in an assembled hydrogel film (Li et al., 2020). Cells localized in this manner could receive electronic signals (hydrogen peroxide) and then transmit their “message” to cells outside of the film through signal synthesis, secretion, and transport to cells occupying the liquid proximal to the electrode and beyond (Li et al., 2020). For affixed cells, instead of randomizing edges at time steps, we fixed edges and maintained them throughout. This mimics a static system, representative of a biofilm (Li et al., 2007; Cornell et al., 2020) or a set of cells localized on an electrode (Terrell et al., 2021).

To quantify structural variation that emerges due to growth and edge dynamics, we used modularity (Newman and Girvan, 2004; Newman et al., 2006; Blondel et al., 2008) as a measure of network structure (Supplementary Figure S4A). In general, modularity describes how well a network is partitioned into various sub-communities (Newman et al., 2006). A single community wherein the connections are near random is represented by a modularity value of zero, while a network where all edges fall within the same community would have a modularity of 1 due to its strong community structure (Newman and Girvan, 2004). For our calculations, we use the Louvain method to calculate the modularity as it is computationally efficient in finding high modularity partitions of large networks (Blondel et al., 2008).

In Figure 6B, we depict the calculated Louvain modularity at 180 timesteps for the cases above (chemical and electrode induction for mixed cultures), as well as the new case where transmitter cells are fixed to the electrode (initial 10 nodes) and the receiver cells are not fixed. For the Transmitters fixed onto the electrode, dividing nodes inherit the edges from their parent nodes without further edge randomization. As expected, our results show that there was increased modularity calculated in the case where some cells are fixed (Transmitters) and some are free to move (Receivers). In general, we found that the modularity of randomized networks was lower than static networks (see Supplementary Figure S4B for simulations of completely fixed systems, not shown here). This is understandable because randomized distribution of edges among the nodes yields an unorganized network structure. In comparison, as static networks grow, they maintain structure.

We further ran simulations with fixed edges for different charge durations and hydrogen peroxide concentrations as in the earlier simulations, to examine static biofilm cultures relative to well stirred systems. We found differences in charge duration and initial hydrogen peroxide concentrations did not affect the modularity as molecular concentrations that are represented by node weights do not affect the spatial structure of the network. We then analyzed the output (i.e., GFP level) for these simulations. To compare the output of these static cultures we calculated the ratio of average GFP at 180 timesteps from randomized networks to the static networks. We use this as a way to measure the benefit of cells in the traditional well-mixed system to those in a fixed or partially fixed system (i.e., cells fixed to an electrode propagating signals to those in fluid nearby). In Figure 6C, we plotted these ratios for each inducer and system type. For the new case of transmitters fixed to an electrode, we also tested a case in which the receivers are also fixed to emulate multilayer deposition of cells onto an electrode as a potential design. This is more representative to a complete biofilm. The fold change calculated from these transmitter fixed cases were done relative to static networks of electrically induced Transmitter/Receiver simulations.

Here we see that for chemical addition, there was little difference between the network structures. This results from the fact that all nodes experience the same initial inducer concentrations. For electrically induced systems, there was minimal effect on the Monoculture at all charge durations. In the Transmitter/Receiver system, we found that for 30 steps of charge there was an approximately 3-fold increase in signal when randomizing the network. In fixed transmitter simulations, we found a substantially larger range for the overall system output. These fold increases are indicative of how edge randomization generally increases output while strategic spatial arrangement of the co-culture with respect to inducer sources can largely amplify signal throughput.

Finally, we assessed how topological effects leading to increased modularity affect signaling within the network. We calculated the percentage of cells that are active (GFP above a 10^3^ threshold) and the median GFP for these Transmitter/Receiver simulations with various edge dynamics (Figures 6D, E). We observed that the static networks had both the lowest median GFP and the fraction of active cells (Figures 6D, E). Interestingly, our simulations suggest that introducing transmitters that are fixed to the electrode increases the overall activation and median GFP over completely randomized networks, and this is irrespective of receiver conformation (fixed or not). We suggest this is due to the faster and increased AI-1 production that is enabled by transmitter proximity to the electrode (Supplementary Figure S4C). Randomizing the receivers further increased estimated output. This is a consequence of allowing the whole receiver population’s increased contact with the transmitter population, as the AI-1 source. This was evident as the network’s GFP distributions where increasing static network components correlate to a wider range in GFP values (Supplementary Figure S4D). Overall, these results reveal that while high modularity yields increased signal heterogeneity, it also lowered signal output compared to low modularity networks. That said, the strategic or intentional organization of subpopulations can drastically increase output, despite increased modularity.

## 4 Discussion

In this work, we developed a graphical network approach for modeling multi-population bacterial cultures. By coarse graining the cell-to-cell signaling interactions that are known to occur in complex bacterial systems (Waters and Bassler, 2005) and leveraging intrinsic network properties that attempt to simulate spatial distributions, we have elucidated signal dynamics that would be very difficult to ascertain using traditional deterministic population scale multicellular modeling. The implementation of a graph-based model allowed us to vary network structures that we had previously implemented experimentally. We were able to determine network parameters (probabilities of growth, molecule production, gene activation) that when employed in the model, accurately recapitulated the experimental observations. Then node weights (other state variables such as inducer levels, substrate levels, etc.) were examined to better understand the experimental results. Perhaps more importantly, with this agreement we then tested hypotheses regarding the spatial composition of microbial systems. Further, by implementing various edge architectures, we attempted to mimic various engineered and endogenous culture structures. We mimicked stirred batch conditions common to biomanufacturing settings via edge randomization. Static edge conformations imitate biofilms found in nature and other immobilized or hydrogel-assembled cell systems. Additionally, we could easily accommodate varied edge profiles in our model so that we could test how relative spatial structures affect communication between different populations.

Owing to the natural tendency to think in terms of subpopulations and quorum sensing (Servinsky et al., 2016), we introduced the notion that network modularity would be a valuable tool in analyzing bacterial networks when organized in the various experimental configurations. In testing fixed spatial conformations we found that for increased modularity, meaning more subcommunities in the network, maximum signal throughput is reduced and delayed for simulations with an electrode as an input source. We suggest this is attributed to the need for the input signal to diffuse into each subcommunity for the secondary signal to then be produced and diffused back out for further signaling. We suggest this introduces an increase in noise at each step of signal transmission due to structural constraints. That said, these decreases in signal can be overcome by spatially orienting transmitter nodes close to the electrode as the input signal source. We further tested fixing all transmitter nodes to the input signal source (the electrode) and found that regardless of whether the receivers were fixed or randomized this restored signal in fixed networks and resulted in higher expression than in randomized simulations. Correspondingly, in Terrell et al. (2021), they demonstrated that by fixing microbes to a gold electrode they could produce AI-1 with electrochemical stimulation, and this was shown to be quite successful in signal propagation (more so than in VanArsdale et al. (2022), where the transmitter and receiver populations were fully mixed in a stirred vessel). Unfortunately, in neither case was it experimentally feasible to monitor the AI-1 diffusion and activation across the system boundaries (Terrell et al., 2021). Here, our work may provide theoretical insight into the signaling occurring in these types of experimental configurations and those found in natural biofilm systems, where measurements in real time and at small distances is difficult.

Additionally, we suggest that models such as this can be further extended to simulate other spatial conformations of cell populations to provide insight into how much input and signal transmission is necessary for successful outcomes (Chun et al., 2021). These include cases where synthetic assembled consortia of higher complexity may be cultured together in batch or spatially fixed within gels (Luo and Shoichet, 2004), between membranes or 3D printed niches (Duraj-Thatte et al., 2021), or within varying ecological niches (Li et al., 2007; Schiessl et al., 2019; Cornell et al., 2020; Ciccarese et al., 2022; Evans et al., 2023). For example, the field of biomaterials has implemented the spatial confinement of cells within hydrogel structures and microcapsules for the use in generating functional living materials and to recreate micro communities found in nature (Dsouza et al., 2022; Molinari et al., 2022; Wang et al., 2022; Yanamandra et al., 2022).

## Data Availability

The simulation datasets for this study can be found here - http://github.com/kaychun29/bio-network-simulations. Further inquiries can be made to the corresponding author - bentley@umd.edu.
