# Supplementary material for: Assessing electrogenetic activation via a network model of biological signal propagation
Source: Front Syst Biol. 2024 Mar 1;4:1291293. doi: 10.3389/fsysb.2024.1291293 (PMC12342027; doi:10.3389/fsysb.2024.1291293)
Supplement: Supplementary file 1 [file Table1.DOCX]

| **Parameters** | **Values** | **Descriptions** |
| --- | --- | --- |
| $s_{0}$ | 20 | Initial substrate weight assigned to each node. |
| $P_{div}$ | 0.015 | Probability of division at each timestep for each node. |
| $\alpha$ | 1 | Diffusion coefficient, generalized to apply to all signaling molecules in the network. |
| $\Delta t$ | 0.01 | The timestep size by which diffusion across edges occurs. |

**Supplemental Table 1.** Simulation parameters with corresponding values used throughout the manuscript (unless noted otherwise) and descriptions.


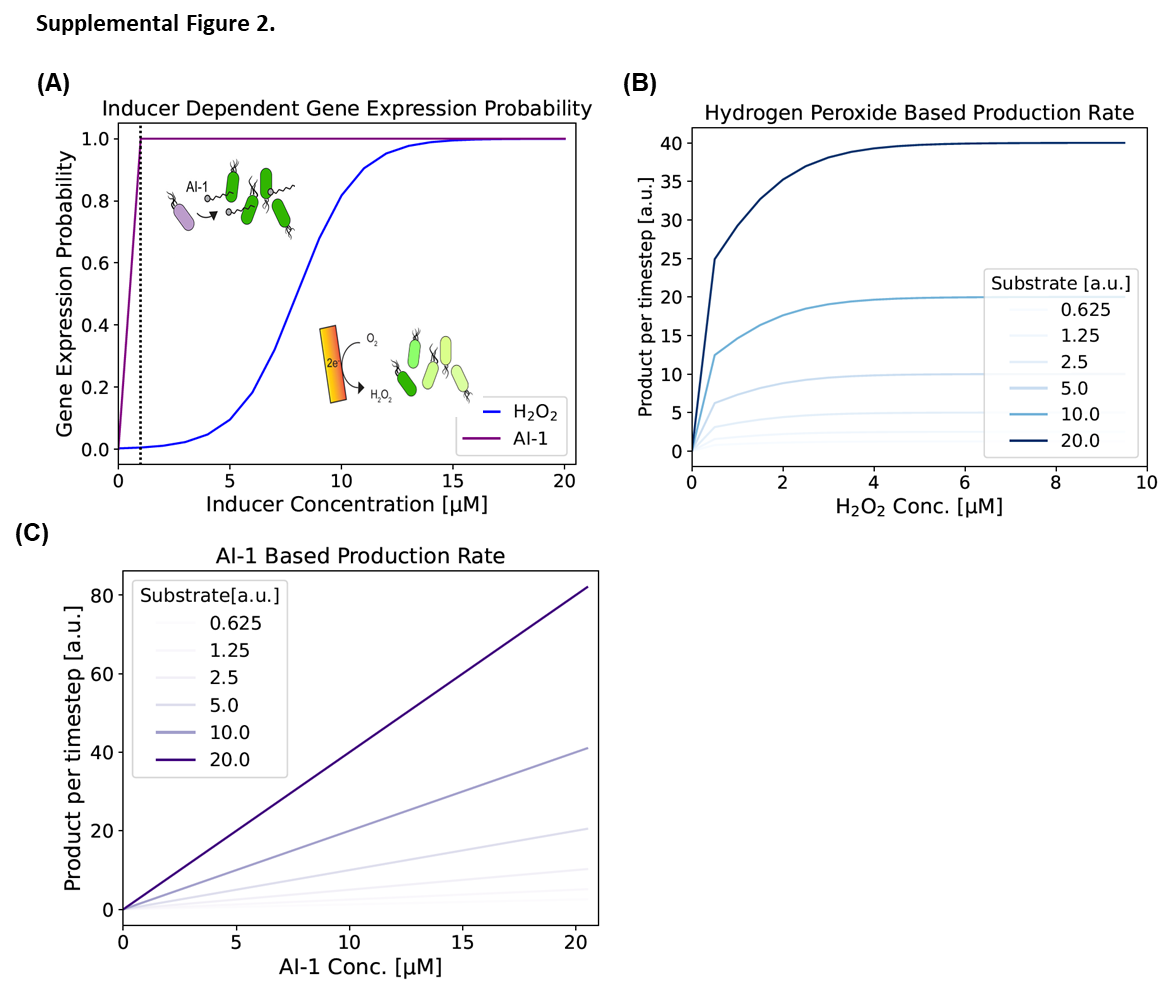


**Supplemental Figure 1. Gene activation and molecular production. (A)** The gene activation probability for AI-1(purple) and hydrogen peroxide (blue) inducible targets dependent on inducer concentration. **(B)** Hydrogen peroxide and substrate dependent molecular production rate for various substrate concentrations. **(C)**  AI-1 and substrate dependent molecular production rate for various substrate concentrations.

We show in **Supplemental Figure 1A**, two emperical relationships for modulating gene expression as a function of inducer. These functions are applied at each time step for each node. The hydrogen peroxide and AI-1 inducible and substrate dependent production rates are depicted in **Supplemental** **Figure 1B and 1C**, respectively.


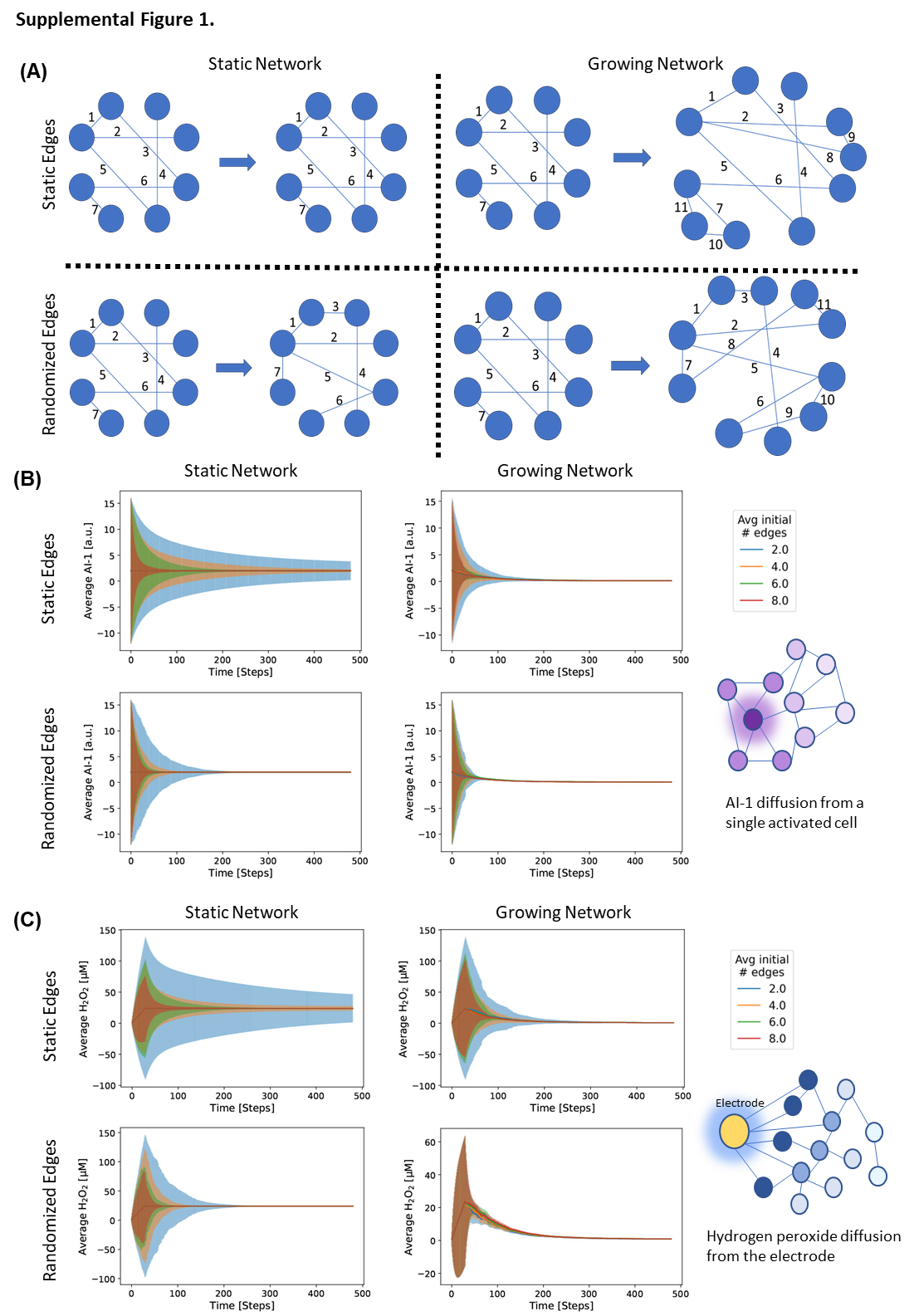


**Supplemental Figure 2.** **Diffusion schematic and dynamics. (A)** Schematic of edge dynamics for static and randomized edge properties for both static and growing networks. The transition arrows (blue) represent a time step. The static system has no change in nodes or edges. The growing network shows the doubling of one cell node and subsequent edge placement. Randomized edge networks demonstrate random edge rearrangement across time. **(B)** Average AI-1 of all nodes plotted over time for a static network, with static edges, a growing network with static edges, a static network with randomized edges, and a growing network with randomized edges. Shaded regions are error bars that indicate standard deviation of AI-1 for all nodes in the network. The schematic on the right depicts a graph with AI-1 diffusing from a single node in dark purple, with high saturation correlating to high concentration. **(C)** Average hydrogen peroxide level of all nodes plotted over time for a static network, with static edges, a growing network with static edges, a static network with randomized edges, and a growing network with randomized edges. Error bars (appear as shaded regions) indicate standard deviation of hydrogen peroxide for all nodes in the network. The schematic on the right depicts a graph with hydrogen peroxide diffusing from an electrode node with a high saturation of blue correlating to high concentration.

Here, we demonstrate the effects that the inducer source, edge density, growth, and edge randomization have on diffusion of signaling molecules throughout the network. We simulated two base cases to demonstrate these effects: one where inducers may come from a highly concentrated source node and one where an electrode may generate inducers at its surface (**Supplemental Figure 2)**. We assess the distribution of the signal across the network for cases of fixed, randomized, and growing networks, as depicted in **Supplemental Figure 2A**. That is, we have included cases of non-growth as well as growth to reflect information transfer within slow growing populations and faster growing populations. Naturally, our simulation data is heterogeneous; this allows for the creation of different trajectories that span wide phenotypic ranges that could be considered when designing systems.

In **Supplemental Figure 2B**, we depict simulations for a point source scenario; this would be representative of a single cell releasing a single burst of signaling molecules followed by its dispersion. We then plotted the average and standard deviation of the AI-1 signal over time, showing that the standard deviation approaches zero as concentrations equilibrate across the network. Notably, randomized edges engender rapid diffusion across the network irrespective of static or growing population dynamics. When combining both network growth and edge randomization we see the fastest concentration convergence out of the four cases presented. We also tested varying initial edge densities (colors in plots) and found that increasing initial edge density allows for faster decrease in standard deviation. We also note that in the cases of network growth or edge randomization the standard deviation will approach zero, irrespective of the initial edge density with the exception of static networks with low (~2) initial edge densities. In this case, the network had not been fully connected by 480 timesteps and therefore, the AI-1 would not be able to diffuse across the whole network. This situation is perhaps indicative of two separate communities of low-density biofilms.

Interestingly, in the case where hydrogen peroxide is produced at an electrode, we found similar results. In **Supplemental Figure 2C**, we depict results from a network containing a single electrode node which produced 40 µM of hydrogen peroxide per timestep for 30 steps. Compared to diffusion from an already highly concentrated node as in **Supplemental Figure 2B,** we observed here that electrode-generated hydrogen peroxide resulted in a delayed onset as well as a delayed convergence. This was particularly true for the simulations of static networks. We suggest that a delay in convergence was not as evident in growing networks due to the increase in density of nodes connected to the electrode; this allows for more rapid spread across the network. Also, in all growing networks, the signaling molecules were continually split between daughter nodes so that their concentration continually decreased over time.


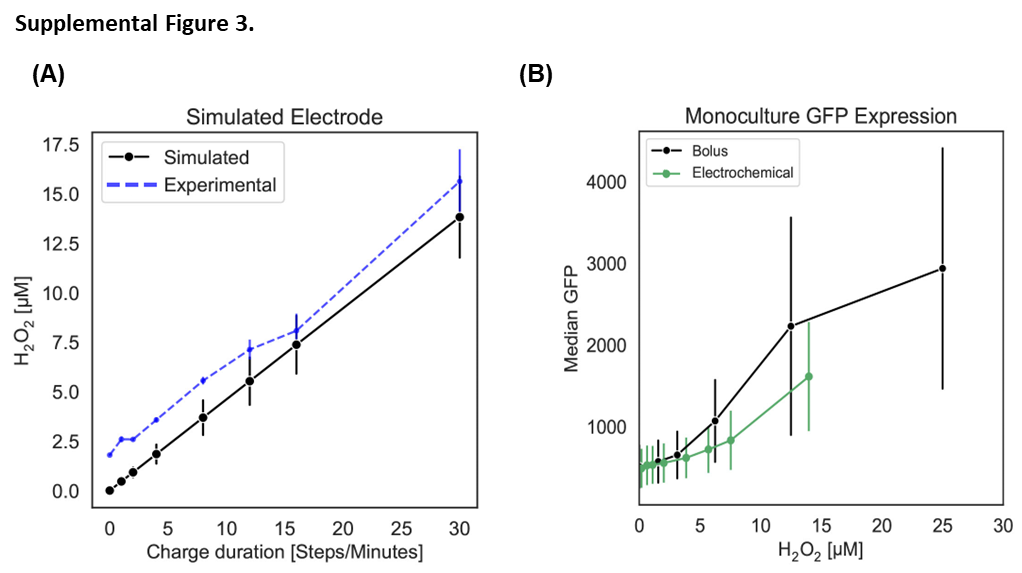


**Supplemental Figure 3.** **Electrode characteristics.** **(A)** Average hydrogen peroxide amongst an 100 node network dependent on charge duration (black) alongside experimentally measured hydrogen peroxide at various charge durations at -0.55V vs Ag/AgCl on a 2 mm gold disk electrode. Error bars in blue represent standard deviation of experimental results (n=3)^1^. Bars in black represent standard error for the simulated electrode (n=10). **(B)** Median GFP of the Monoculture Network at 180 timesteps post induction, with chemical induction (black) and electrochemical induction (green). Bars represent the standard deviation of a network data consisting of 10 simulation replicates.

The experimental conditions yield a linear correlation between the hydrogen peroxide generated and charge, as shown in **Supplemental Figure 3A** in blue^8^. In black**,** we demonstrate that with the initial network size of 100 and a 46µM hydrogen peroxide per timestep production rate the average hydrogen peroxide concentration across the network fits with experimental results. We note that in our simulations we subtracted the background in our simulated electrode fit, such that at time zero no hydrogen peroxide is present in the network compared to experimental results in which 2.5 µM of hydrogen peroxide was measured without electrical stimulation (**Supplemental Figure 3A**). In **Supplemental Figure 3B**, we depict the Monoculture response for chemically and electrically induced simulations. We simulated electrochemical induction for various charge durations and plotted the median GFP expression at 180 steps post applied charge against the measured average hydrogen peroxide at the end of the charge duration. Here, the chemical addition increased more rapidly with H_2_O_2_, compared to the electrode generated case where it takes time for diffusion to activate nodes throughout the network. In effect, this dampens the signal, resulting in lower GFP output for the same average hydrogen peroxide input.


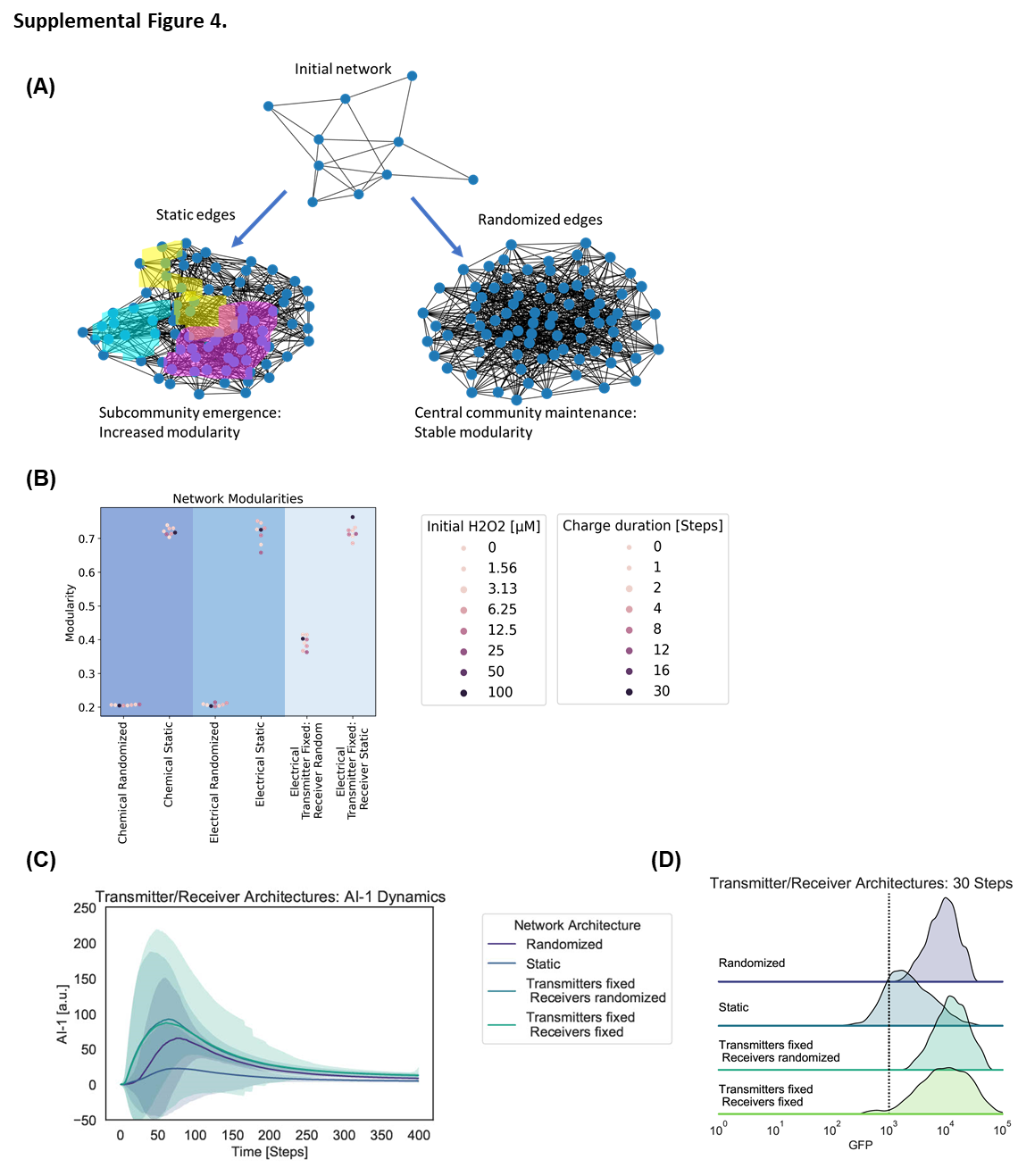


**Supplemental Figure 4.** **Signal transmission measures for various Transmitter/Receiver network architectures.** **(A)** Graph schematics depicting network growth with and without edge randomization, showing subcommunity emergence and modularity increase in static edge networks compared to randomized edge networks. **(B)** Network modularity of differing node arrangements and edge dynamics, including fully static edges, at timestep 180. **(C)** AI-1 average across entire network over time for various Transmitter/Receiver network architectures, shadows represent standard deviation of aggregated network data from 10 simulation replicates. **(D)** The GFP distribution for each Transmitter/Receiver architecture tested at time 180 steps, induced with a charge duration of 30 steps. Distributions are representative of aggregated network data from 10 simulation replicates.

In **Supplemental** **Figure 4A**, we visualize the effect of edge randomization on a network. Without edge randomization, subcommunities arise in the network and modularity is therefore increased. We observe this effect in the distribution of the signaling molecule AI-1 with various network structures in **Supplemental Figure 4B**. In the static network AI-1 production was lower than in the randomized network. This suggests that the delay caused by the sequential chain of signal diffusion across a static network from electrode to transmitter to receiver reduces the activation range of the system. In the two fixed transmitter networks, the average AI-1 output is similar, but the standard deviation for the system with fixed receivers was larger than with randomized receivers. This reflects the higher AI-1 output for both cases in which transmitters are fixed onto the electrode and the increased heterogeneity of highly modular static networks. We additionally plotted the GFP distributions for these networks in **Supplemental Figure 4C**. Here the fixed transmitters and fixed receivers exhibited the greatest spread in GFP output. The networks in which the receivers are static have a wider distribution compared to those with randomized receiver populations.

**Runtime and complexity**

We suggest models such as ours are suitable to adapt to these systems of increased complexity, both in terms of architecture flexibility and computationally. We found that when simulating systems previously described with a 5-fold increased initial network size (500 nodes), our runtimes were approximately 3 hours compared to 20 minutes for an initial 100 nodes. It appeared that runtimes were primarily influenced by network size, such that including a larger number of subpopulations to model complex consortia is certainly feasible. A model such as this can be adapted to these orientations to simply test and interrogate how different topologies, in addition to various signaling modalities, may affect signal throughput and subsequent function.
